# AtBBX29 integrates photomorphogenesis and defense responses in *Arabidopsis*

**DOI:** 10.1007/s43630-023-00391-8

**Published:** 2023-02-18

**Authors:** Ana L. Medina-Fraga, Lucas A. Chinen, Patricia V. Demkura, Micaela Z. Lichy, Jonathan Gershenzon, Carlos L. Ballaré, Carlos D. Crocco

**Affiliations:** 1 Facultad de Agronomía, IFEVA, Consejo Nacional de Investigaciones Científicas y Técnicas-Universidad de Buenos Aires, Av. San Martín 4453, C1417DSE Ciudad Autónoma de Buenos Aires, Argentina; 2 Department of Biochemistry, Max Planck Institute for Chemical Ecology, Jena, Germany; 3 IIBIO, Consejo Nacional de Investigaciones Científicas y Técnicas-Universidad Nacional de San Martín, B1650HMP Buenos Aires, Argentina; 4 Department of Plant Sciences, Section of Biology, Faculty of Sciences, University of Geneva, 1211 Geneva 4, Switzerland

**Keywords:** BBX, Plant photomorphogenesis, Plant defense response, MYB, UV-B

## Abstract

Light is an environmental signal that modulates plant defenses against attackers. Recent research has focused on the effects of light on defense hormone signaling; however, the connections between light signaling pathways and the biosynthesis of specialized metabolites involved in plant defense have been relatively unexplored. Here, we show that *Arabidopsis* BBX29, a protein that belongs to the B-Box transcription factor (TF) family, integrates photomorphogenic signaling with defense responses by promoting flavonoid, sinapate and glucosinolate accumulation in *Arabidopsis* leaves. *AtBBX29* transcript levels were up regulated by light, through photoreceptor signaling pathways. Genetic evidence indicated that AtBBX29 up-regulates *MYB12* gene expression, a TF known to induce genes related to flavonoid biosynthesis in a light-dependent manner, and *MYB34* and *MYB51*, which encode TFs involved in the regulation of glucosinolate biosynthesis. Thus, *bbx29* knockout mutants displayed low expression levels of key genes of the flavonoid biosynthetic pathway, and the opposite was true in *BBX29* overexpression lines. In agreement with the transcriptomic data, *bbx29* mutant plants accumulated lower levels of kaempferol glucosides, sinapoyl malate, indol-3-ylmethyl glucosinolate (I3M), 4-methylsulfinylbutyl glucosinolate (4MSOB) and 3-methylthiopropyl glucosinolate (3MSP) in rosette leaves compared to the wild-type, and showed increased susceptibility to the necrotrophic fungus *Botrytis cinerea* and to the herbivore *Spodoptera frugiperda*. In contrast, *BBX29* overexpressing plants displayed increased resistance to both attackers. In addition, we found that AtBBX29 plays an important role in mediating the effects of ultraviolet-B (UV-B) radiation on plant defense against *B. cinerea*. Taken together, these results suggest that AtBBX29 orchestrates the accumulation of specific light-induced metabolites and regulates *Arabidopsis* resistance against pathogens and herbivores.

**Graphical Abstract:**

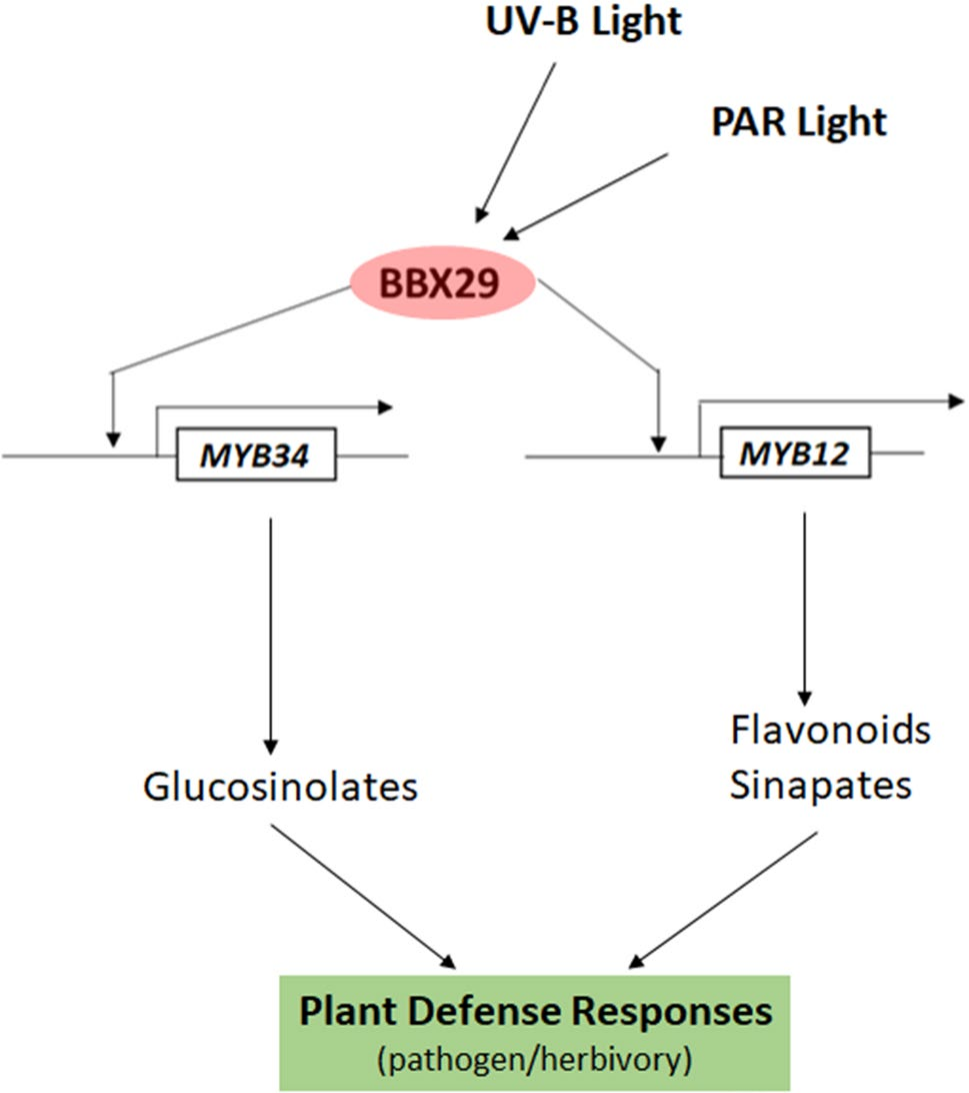

**Supplementary Information:**

The online version contains supplementary material available at 10.1007/s43630-023-00391-8.

## Introduction

Plants cope with abiotic and biotic stresses by activating adaptive physiological and morphological responses. Some of these plastic responses appear to be effective against multiple stresses, suggesting that different environmental signals can converge in the regulation of similar cellular pathways. The accumulation of specialized metabolites in leaves is a good example of this convergence. For example, anthocyanins, carotenoids, flavonoids and glucosinolates (GS) can be induced by a large number of environmental factors, including low temperature, high light, ultraviolet (UV) radiation, pathogen attack, wounding, and nutritional stress, among others, and can provide protection against multiple stressors [1–3].

Light is one of the environmental factors that has a strong effect on the accumulation of specialized metabolites in leaves, acting through photomorphogenetic signaling pathways that are activated by photoreceptors, such as the red- and far-red light-sensing phytochromes, the blue/UV-A-perceiving cryptochromes and phototropins, and the UV-B-sensing photoreceptor UVR8 [4, 5]. The action of photoreceptors depends on a set of TFs that belong to different families, such as bZIPs, MYBs and BBXs, which orchestrate the transcriptional responses to changes in the light environment [6–8]. In *Arabidopsis* seedlings, HY5 (ELONGATED HYPOCOTYL 5) forms a central hub downstream of all photoreceptors, to induce the transcriptional activation of *MYB* TFs that regulate the biosynthesis of phenolic compounds. These TF include MYB11, MYB12 and MYB111 for flavonol, MYB123 for proanthocyanidin and MYB75/PAP1 for proanthocyanin biosynthesis [9–11]. Downstream of HY5 and MYBs, light-related factors establish a coordinated action to activate the transcription of genes of the phenylpropanoid biosynthetic pathway, including *CHALCONE SYNTHASE (CHS), FLAVONOL SYNTHASE (FLS), FLAVANONE 3-HYDROXYLASE (F3H)* and *CHALCONE ISOMERASE (CHI)* [12, 13].

The involvement of BBX proteins in the photoregulation of specialized metabolism has been studied in *Arabidopsis* seedlings, leading to the description of possible models of BBX action. In a general model, BBXs appear as potential partners for HY5 to coordinate and modulate its specificity and activity in the transcriptional regulation of photomorphogenic genes, including some MYB TFs and genes belonging to the flavonoid biosynthetic pathway [14, 15]. For example, AtBBX21 and AtBBX22 interact with HY5 and enhance its biochemical activity leading to increased expression of flavonoid biosynthesis genes and accumulation of anthocyanins [15–17]. AtBBX23 plays a positive role in the control of anthocyanin accumulation by binding to the *CHS* promoter region in a HY5-dependent manner [18]. In contrast, AtBBX24, AtBBX25 and AtBBX32 negatively control anthocyanin accumulation likely by inhibiting HY5 activity and the transcription of HY5-activated genes [19–21]. Beyond this model, some BBXs have been reported to directly associate with promoters of various flavonoid biosynthesis genes to alter their expression in an HY5-independent manner. For example, AtBBX21 and AtBBX22 physically bind to the promoter of *CHI* and activate *CHI* transcription [15].

In contrast to the situation in seedlings, the mechanisms by which *Arabidopsis* BBXs regulate the light-induced accumulation of specialized metabolites in rosette leaves is not well established. There is evidence from studies carried out in various crop species that indicate an involvement of BBXs in the photoregulation of flavonoid accumulation. In poplar, PtBBX23 directly binds to the promoter regions of proanthocyanidin and anthocyanin-specific genes to enhance their transcription [22]. In rice, anthocyanin biosynthesis is induced and fine-tuned by OsBBX14 [23]. In pear, PpBBX16 and PpBBX18 antagonistically regulate light-induced anthocyanin accumulation in the fruit [24]. In apple, MdBBX33 and MdBBX37 have been reported to influence anthocyanin accumulation in a light-dependent manner [25, 26]. Heterologous expression of *AtBBX21* in potato plants induced the expression of phenylpropanoid biosynthesis genes and showed a higher production of anthocyanins and phenolic compounds [27]. These results suggest a central role of BBX proteins in the regulation of specialized metabolism in green plants.

It has been demonstrated that light-regulated metabolites like flavonoids, sinapates and GSs are functionally important in plant defense against a variety of attackers [28–32]. In several species, low Red/Far-Red ratios (a signal of shading and plant-plant competition) promote plant susceptibility via the inactivation of phytochrome B, which leads to reduced accumulation of phenylpropanoids and GSs in leaves and attenuation of JA-mediated responses [33–35]. In contrast, UV-B radiation (which is associated with open habitats and canopy gaps) can enhance plant defense against pathogens and herbivores via JA-dependent and JA-independent mechanisms [36, 37]. In *Arabidopsis*, physiological doses of UV-B radiation promote the accumulation of phenolic compounds, including flavonoids and sinapates, in a JA-independent manner [30]. Furthermore, at least part of the effect of UV-B radiation increasing plant resistance to the fungus *Botrytis cinerea* is mediated by the UVR8 photoreceptor via stimulation of sinapate biosynthesis [30]. GSs also play a main role as defensive metabolites in the Brassicaceae family, where the accumulation of GSs negatively affects the growth and development of some herbivores and mediates plant antifungal defenses [31, 38–40]. It has been shown that light could modulate the production of GSs vía regulation of GS biosynthetic genes [41, 42] and CONSTITUTIVELY PHOTOMORPHOGENIC 1 (COP1), the key repressor of light signaling, can regulate GS biosynthesis, acting through a mechanism dependent of JA-signaling [32].

Here we show that AtBBX29, a BBX protein that belongs to the structure group V [43] is involved in flavonoid and glucosinolate accumulation in *Arabidopsis* leaves. *AtBBX29* transcription was regulated by light acting through photoreceptors, and the AtBBX29 protein showed nuclear localization. We found that AtBBX29 positively regulated the transcription of several *MYB* genes and genes encoding enzymes involved in the biosynthesis of specific photoprotective compounds. *bbx29* mutant plants were impaired in the accumulation of flavonoids (kaempferol glycosides), sinapates (sinapoyl malate) and some glucosinolates (indol-3-ylmethyl glucosinolate, I3M; 4-methylsulfinylbutyl glucosinolate, 4MSOB and 3-methylthiopropyl glucosinolate 3MSP) in rosette leaves, and this reduced accumulation of defensive metabolites correlated with higher susceptibility to the fungus *B. cinerea* and to an insect herbivore (*Spodoptera frugiperda*). In contrast, *BBX29* overexpressing plants displayed increased resistance to both attackers. Transcripts of *AtBBX29* were also up-regulated by methyl jasmonate (MeJA) treatment in a transient manner, but jasmonate metabolism and response appeared to be functional in *bbx29* mutants. Taken together, these results suggest that AtBBX29 is involved in the photoregulation of the biosynthesis of specialized metabolites that play an important role in *Arabidopsis* defense against attackers.

## Results

### Red, Blue and UV-B light induce *AtBBX29* transcript levels through the photomorphogenic signaling pathways

**Fig. 1:**
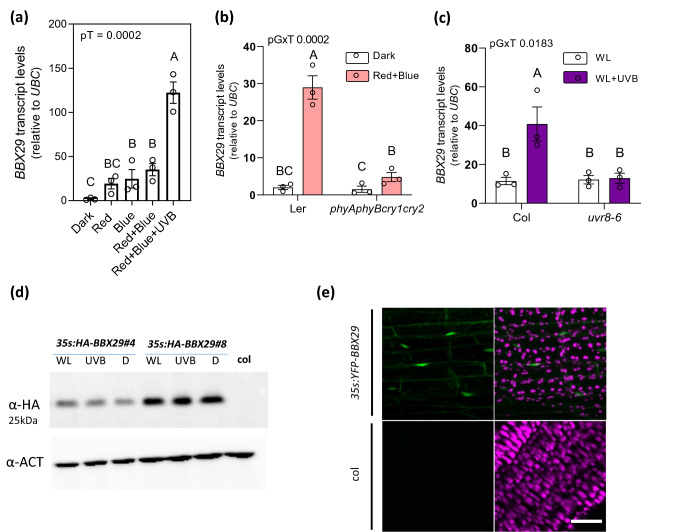
Photomorphogenic regulation of AtBBX29. **a** Transcript levels of *AtBBX29* relative to *UBC* in 5-d-old dark-grown Col seedlings after transfer for 1 h to Red (10 μmol m^−2^ s^−1^; R), Blue (10 μmol m^−2^ s^−1^; B), R + B (10 μmol m^−2^ s^−1^) or R + B light plus weak UV-B supplementation. **b** Transcript levels of *AtBBX29* in 5-d old dark-grown seedlings (Ler and *phyAphyBcry1cry2*) after transfer to 1 h of B + R (10 μmol m^−2^ s^−1^) or kept in darkness (control). **c** Transcript levels of *AtBBX29* in 5-days old seedlings (Col and *uvr8-6*) grown in continuous white light (5 μmol m^−2^ s^−1^) were either supplemented with 1 h of 1 μmol m^−2^ s^−1^ of UV-B radiation (WL + UVB) or kept in white light (WL). Values relatives to *UBC* transcript levels. The P-value for the G (Genotype) x T (treatment) interaction term of the ANOVA is shown; different letters indicate significant differences between means (P < 0.05, LSD Fisher test). Each bar represents the mean ± SEM (n = 3 biological replicates). **d** AtBBX29 protein levels in 5-day-old seedlings kept for 24 h under different light conditions; ACT2 abundance in the same mebrane is used as a loading control. Seedlings were grown under continuous white light (5 μmol m^−2^ s^−1^) before the light treatments: WL = continuous WL (5 μmol m^−2^ s^−1^); UVB = continuous WL + UV-B (1 μmol m^−2^ s^−1^); D = darkness. **e** YFP fluorescence (green) in transgenic hypocotyls expressing a *35:YFP-BBX29* fusion gene. Wild-type (Col) hypocotyls were used as control. Scale bar: 10 µm. The right panel shows chlorophyll autofluorescence of plastids (purple)

**Fig. 2:**
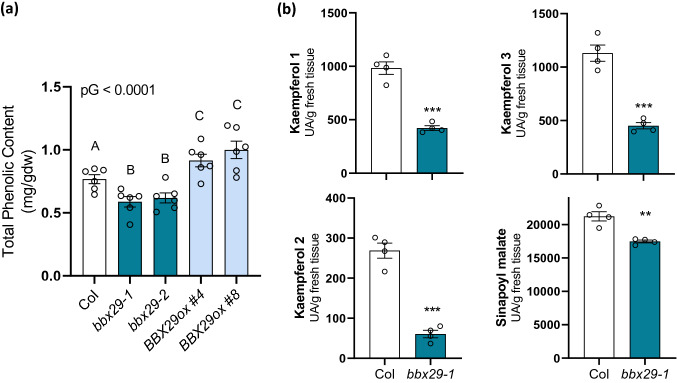
AtBBX29 promotes the accumulation of soluble leaf phenolics.** a** Quantification of total soluble phenolic compounds in rosette leaves of Col, *bbx29-1*, *bbx29-2* and two independent overexpression lines (*BBX29ox*). Data were analyzed by one-way ANOVA. The P-value for the genotype term of the ANOVA is shown (pG); different letters indicate significant differences between means (P < 0.05, LSD Fisher test) and each bar represents the mean ± SEM (n = 6 biological replicates).** b** HPLC quantification of metabolites derived from the phenylpropanoid pathway in rosette leaves (Kaempferol 1, 2 & 3 and sinapoyl malate). Data were analyzed by Student’s t tests, and asterisks indicate a significant difference between Col and mutant lines (*P < 0.05, **P < 0.01, NS, not significant). Each bar represents the mean ± SEM (n = 4 biological replicates)

Previous work revealed a molecular role of AtBBX29 in the regulation of seedling photomorphogenesis, being part of a feedback loop with AtBBX28, AtBBX30, AtBBX31 and HY5 to control light-responsive genes at the transcriptional levels [44]. However, how light regulates AtBBX29 is not clear. To gain further insight into the photomorphogenic regulation of *AtBBX29* transcription, we performed quantitative PCR (RT-qPCR) experiments with 5-d-old wild-type (Col) etiolated seedlings that were transferred from darkness to either 1 h of Red light (R), Blue light (B), R + B or R + B supplemented with weak UV-B light (Fig. 1a). The expression levels of *AtBBX29* were up-regulated by B, R and UV-B light, suggesting that *AtBBX29* transcription may be activated by phytochromes, cryptochromes and UVR8. To corroborate this hypothesis, we first measured *AtBBX29* transcript abundance in wild-type and quadruple *phyA phyB cry1 cry2* mutant seedlings when they were shifted from darkness to R + B light conditions (Fig. 2b); second, we quantified *AtBBX29* transcript levels in wild-type and *uvr8-6* seedlings grown under continuous white light (WL) with or without 1 h of UV-B supplementation. The induction of *AtBBX29* by visible light depended on phytochromes and/or cryptochromes and the effect of additional UV-B radiation required UVR8 (Fig. 1b, c). Given that some BBXs are regulated post-translationally by protein stabilization or degradation [45, 46], we performed an experiment to assess the stability of AtBBX29 under different light conditions. Plants overexpressing *AtBBX29* with the HA tag fused to the N terminus (*35S::HA-BBX29*) in the Col background were cultivated for 5 d under continuous WL and then transferred for 24 h to either WL + weak UV-B or darkness (D). Control seedlings were kept under WL. The results showed that AtBBX29 protein stability was not affected by light under these conditions (Fig. 1d). BBX proteins function as TFs or as cofactors to modulate transcriptional responses in *Arabidopsis* and, therefore, they are expected to be located in the nucleus to exert their regulatory action. To determine the subcellular localization of the AtBBX29 protein, we generated *Arabidopsis* transgenic lines tagged with the Yellow Fluorescent Protein (*35S::YFP-BBX29*). The fluorescence was mainly observed in the nucleus of hypocotyl cells (Fig. 1e).

### AtBBX29 positively regulates the accumulation of soluble phenolic compounds

**Fig. 3:**
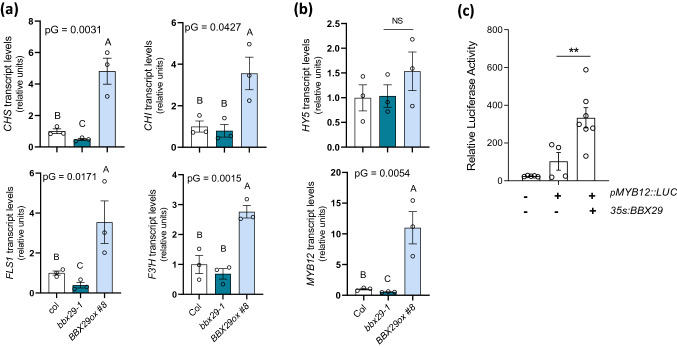
AtBBX29 is a positive transcriptional regulator of genes involved in flavonoid biosynthesis.** a** Expression levels of genes involved in flavonoid biosynthesis (*CHS*, *CHI*, *F3´H* and *FLS1*).** b** Expression levels of transcription factors known to regulate genes involved in flavonoid biosynthesis (*HY5*, *MYB12*). Values are normalized to *IPP2* transcript levels and standardized to Col expression levels. The P-value for the genotype term of the ANOVA is shown (pG) and different letters indicate significant differences between means (P < 0.05, LSD Fisher test). Each bar represents the mean ± SEM (n = 3 biological replicates).** c** Tansient luciferase assay in tobacco leaves. The firefly luciferase gene driven by the *MYB12* promoter (*pMYB12::LUC*) was used as the reporter and *AtBBX29* driven by the 35S CaMV promoter was used as the effector. Empty vectors were used for the effector control (see methods). The values represent the ratio between firefly luciferase activity and renilla luciferase activity. Data were analyzed by one-way ANOVA (**P < 0.01, NS, not significant). Each bar represents the mean ± SEM (n ≥ 4 individual plants)

Some *Arabidopsis* BBXs proteins regulate the light-dependent accumulation of photoprotective flavonoid and phenolic compounds in leaves. In part, this modulation is exerted through the transcriptional regulation of genes involved in flavonoid biosynthesis, such as *CHS*, *CHI*, *FLS1* and others [24, 27, 47, 48]. To investigate the role of AtBBX29, we quantified the accumulation of total soluble phenolic compounds in leaves of 4-week-old plants of wild-type (Col), a T-DNA knockout mutant (*bbx29-1*), a T-DNA knockdown mutant (*bbx29-2)* and two independent overexpression lines (BBX29ox#4 and BBX29ox#8; SI Fig. S1). Plants were grown under controlled environmental conditions (10 h light/14 h dark) with 110 μmol m^−2^ s^−1^ of photosynthetically active radiation (PAR). Mutant plants had significantly less total phenolic compounds compared to Col plants, whereas *BBX29ox* lines presented higher content of total leaf phenolics (Fig. 2a). Next, we analyzed the pool of soluble phenolic compounds by HPLC in *bbx29-1* mutants and Col leaves. We found that *bbx29-1* leaves had significantly less accumulation of kaempferols and sinapoyl malate than wild-type leaves (Fig. 2b), suggesting that AtBBX29 plays a role in the biosynthesis of these photoprotective metabolites. Key genes of the flavonoid biosynthetic pathway, *CHS, FLS1, CHI* and *F3´H* transcripts were up-regulated when *AtBBX29* was over-expressed in *Arabidopsis*, and *CHS* and *FLS* were significantly down-regulated in the *bbx29-1* knockout mutant compared to Col plants (Fig. 3a).

The flavonoid biosynthetic pathway is transcriptionally controlled by a network of transcription factors, where HY5 together with MYB12, MYB11 and MYB111 are involved in the photoregulation of these genes by binding to their promoters through different cis-elements [11, 49, 50]. Some BBX proteins have the ability to bind to the promoter regions of *MYBs* [24, 48] and *HY5* [8, 17, 51] gene to activate their transcription. To investigate whether AtBBX29 plays a role in the regulation of these TFs, we evaluated the expression levels of *MYB12, MYB11, MYB111* and *HY5* in *bbx29-1* mutant, *BBX29ox* lines and wild-type (Col) adult plants. We found that *HY5* transcript levels were not affected by *AtBBX29* (Fig. 3b), but *MYB12* transcript levels were down-regulated in the *bbx29-1* mutant and up-regulated in *BBX29ox* plants compared to Col plants (Fig. 3B). *MYB11* and *MYB111* transcripts were only affected in *BBX29ox* plants compared to Col (SI Fig. S2). These results indicate that AtBBX29 is necessary to modulate *MYB12* expression levels. Additionally, we performed a transient expression assay in *Nicotiana benthamiana* leaves, where the firefly luciferase gene driven by the *MYB12* promoter (*pMYB12::LUC*) was used as the reporter and *AtBBX29* driven by the 35S CaMV promoter was used as the effector. We found that the transient expression of *AtBBX29* activates the promoter of *MYB12 *in vivo (Fig. 3c), suggesting that AtBBX29 acts up-stream of MYB12 in the light-responsive mechanisms that control the flavonoid biosynthetic pathway.

### AtBBX29 positively regulates glucosinolate accumulation

**Fig. 4:**
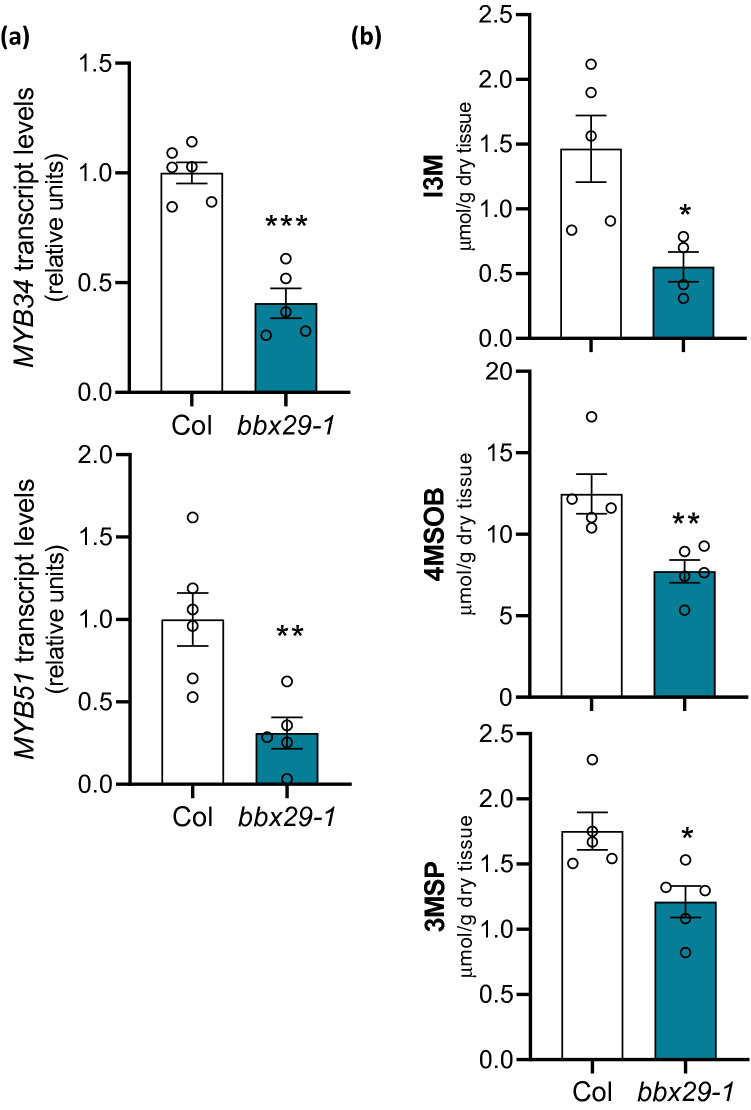
AtBBX29 regulates glucosinolate accumulation in *Arabidopsis* leaves.** a** Expression levels of genes related to the glucosinolate pathway (*MYB34* and *MYB51*) in Col and *bbx29-1* rosette leaves. Values are normalized to *IPP2* transcript levels and standardized to Col expression levels.** b** I3M, 4MSOB and 3MSP accumulation in Col and *bbx29-1* rosette leaves. Data were analyzed by Student’s t tests, and asterisks indicate a significant difference between Col and *bbx29-1* mutant plants (*P < 0.05, **P < 0.01, ***P < 0.001; NS, not significant). Each bar represents the mean ± SEM (n ≥ 5 biological replicates)

Light can modulate the GS accumulation in leaves, contributing to the defensive status of the plant [32, 42, 52, 53]. In *Arabidopsis*, MYB34, MYB51 and MYB122 TFs play an indispensable role in the regulation of GS biosynthesis genes [54]. Given that AtBBX29 regulates light responsive-MYB transcripts (Fig. 3b; SI Fig. S2) we asked whether the MYB-GS-related genes are affected in *bbx29-1* mutant plants and *BBX29ox* lines. We found that AtBBX29 positively regulates *MYB34* and *MYB51* transcription (Fig. 4a; SI Fig. S3) in plants grown under our experimental conditions (short days and 110 μmol m^−2^ s^−1^ of PAR). To evaluate if AtBBX29 plays a role in GS accumulation, we quantified aliphatic and indolic GSs in *Arabidopsis* rosette leaves. We found that *bbx29-1* knockout mutant plants had lower levels of I3M, 4MSOB and 3MSP than wild-type plants (Fig. 4b). The *bbx29-2* knockdown mutants were significantly impaired in I3M accumulation but had normal levels of 4MSOB or 3MSP (SI Fig. S4). Taken together, these results indicate that AtBBX29 positively controls the biosynthesis and accumulation of GSs in *Arabidopsis* leaves.

### AtBBX29 is a positive regulator of *Arabidopsis* defense against *Botrytis cinerea* and *Spodoptera frugiperda*

**Fig. 5:**
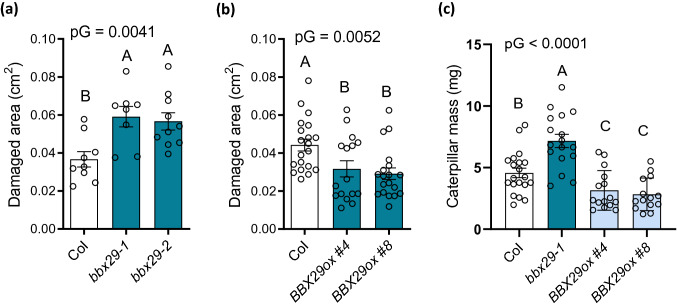
AtBBX29 plays a role in defense against *Botrytis cinerea* and *Spodoptera frugiperda* in *Arabidopsis* leaves.** a** *B.cinerea* bioassay with Col, *bbx29-1* and *bbx29-2* plants.** b** *B. cinerea* bioassay with Col and *BBX29ox* overexpression lines. The bars show the size of the lesions caused by *B. cinerea* on rosette leaves 2 d after inoculation (see methods). Each bar represents the mean ± SEM (n = 10–20 plants) **c ***S. frugiperda* bioassay with Col, *bbx29-1* and *BBX29ox* plants. Bars show the caterpillar mass after 5 day feeding on the indicated genotypes (see methods). Data were analyzed by one-way ANOVA. The P-value for the genotype term of the ANOVA is shown (pG); different letters indicate significant differences between means (P < 0.05, LSD Fisher test). Each bar represents the mean ± SEM (n = 16–22 larvae)

Phenolic and GS compounds play important roles in leaf defense against a variety of attackers. To investigate whether AtBBX29 is involved in defense responses, we performed bioassays with *bbx29* mutants, *BBX29ox* and Col plants challenged with the necrotrophic pathogen *B. cinerea* and the herbivore *S. frugiperda*. *bbx29-1* and *bbx29-2* mutants showed increased susceptibility to the fungus (Fig. 5a) compared to Col plants, whereas *BBX29ox* lines displayed enhanced resistance (Fig. 5b).

In the *Spodoptera* bioassay, the caterpillars that were fed on *bbx29-1* grew faster than those fed on Col plants, and the opposite was true for the caterpillars that consumed *BBX29ox* plants (Fig. 5c). These data indicate that AtBBX29 contributes to *Arabidopsis* defense against *B. cinerea* and *S. frugiperda*.

Given that the AtBBX29 protein can physically interact with HY5 [44] and that, at the seedling stage, HY5 TF activity promotes the expression of *MYB12* and phenylpropanoid biosynthetic genes in a light-dependent manner [11], we asked whether HY5 has a role modulating defense responses in plants at the rosette stage. In the *B. cinerea* bioassay, *hy5* mutants did not display a susceptibility phenotype compared to their corresponding wild-types (SI Fig. S5). These results suggest that the positive effect of AtBBX29 on *Arabidopsis* defense is unlikely to be dependent on HY5 activity.

### AtBBX29 does not affect jasmonate levels

**Fig. 6:**
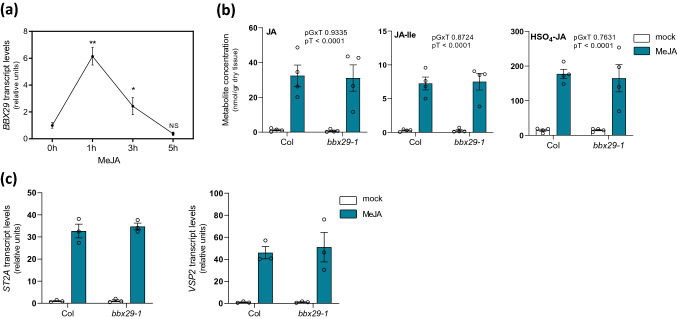
MeJA transiently up-regulates *AtBBX29* but AtBBX29 does not affect JA metabolism or JA signaling pathway.** a** Expression levels of *AtBBX29* in Col plants sprayed with MeJA (50 µM) and harvested at different time points: 0 h (control), 1, 3 or 5 h. Values are normalized to IPP2 transcript levels and expressed relative to 0 h expression levels. Data were analyzed by Student’s t test, and asterisks indicate significant differences between control (0 h) and other time points (*P < 0.05, **P < 0.01, NS, not significant). Each datum point represents the mean ± SEM (n = 3 biological replicates).** b** Quantification of jasmonate pools in Col and *bbx29-1* plants harvested 6 h after treatment with 200 µM MeJA or mock solutions. Each bar represents the mean ± SEM (n = 4 biological replicates; see methods) and the P values for the relevant terms of the ANOVA are shown (two-way ANOVA, G: Genotype, T: hormonal treatment, GxT interaction).** c** Expression levels of JA-markers genes (*ST2a* and *VSP2*). Values are normalized to *IPP2* transcript levels and standardized to Col mock expression levels. Each bar represents the mean ± SEM (n ≥ 3 biological replicates) and the P values for the relevant terms of the ANOVA are shown (two-way ANOVA, G: Genotype, T: hormonal treatment, GxT interaction)

Plant defenses against necrotrophic pathogens and chewing insects are controlled by jasmonates, and *AtBBX29* transcripts were found to be up-regulated by MeJA in microarray data obtained by our group [53]. To investigate potential connections between *AtBBX29* and jasmonic acid (JA) signaling or metabolism, we quantified by qRT-PCR *AtBBX29* transcripts in plants treated with MeJA and measured JA pools in *bbx29-1* and Col plants at the rosette stage. *AtBBX29* transcripts were transiently up-regulated by MeJA (Fig. 6a), which is consistent with our previous microarray data. When Col and *bbx29-1* mutant plants were sprayed with MeJA, they responded with the expected increase in JA pools, including JA, JA-Ile and a sulfated derivative (HSO_4_-JA) whose accumulation has been shown to be regulated by light [53]. The basal levels of these pools and their response to MeJA were not affected in the *bbx29-1* mutant (Fig. 6b). In agreement with the metabolic data, the expression levels of JA-marker genes, *VSP2* and *ST2a*, were not affected in *bbx29-1* mutant plants (Fig. 6c). Collectively, these results suggest that the function of *AtBBX29* in plant resistance against attackers is not related to alterations in JA metabolism or signaling.

### AtBBX29 is required for the protective effect of UV-B radiation on plant resistance to *B. cinerea*

**Fig. 7:**
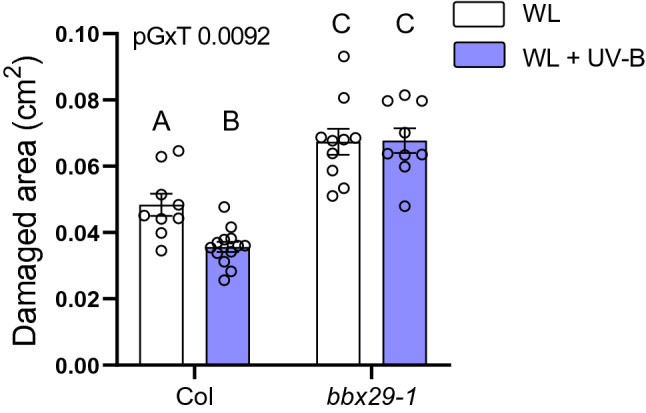
AtBBX29 is required for the effect of UV-B radiation promoting *Arabidopsis* resistance to *B. cinerea. B. cinerea* bioassay in Col and *bbx29-1* plants grown under WL conditions with or without supplementation with 1 μmol m^−2^ s^−1^ of UV-B radiation (see methods)*.* Each bar represents the average size of the lesions caused by *B. cinerea* on rosette leaves 2 d after inoculation ± SEM (n ≥ 9 individual plants). The P-value for the G (Genotype) x T (UV-B treatment) interaction term of the ANOVA is shown; different letters indicate significant differences between means (P < 0.05, LSD Fisher test)

Activation of the UV-B photomorphogenic pathway enhances plant defense responses, where one of the key defensive strategies includes the regulation of specialized metabolites [30, 55]. Under these UV-B-enriched light conditions, sinapate metabolites play an important role in increasing plant resistance against *B. cinerea* [30]. Given that UV-B strongly promotes *AtBBX29* transcript accumulation and that *bbx29-1* mutant plants show reduced sinapate levels, we asked whether AtBBX29 is involved in the effect of UV-B radiation enhancing *Arabidopsis* defenses. To this end, we carried out a *B. cinerea* bioassay with wild-type and *bbx29-1* knockout plants cultivated under white light with or without the addition of low doses of UV-B radiation. As expected [30], low doses of UV-B increased the resistance of Col plants to *B. cinerea*, but this effect was absent in *bbx29-1* plants (Fig. 7).

## Discussion

Light signals are known to modulate the accumulation of defense-related specialized metabolites in leaves [35]. Despite considerable advances in recent years, further work is needed to chart the molecular connections between photoreceptor signaling pathways and defense responses. In this context, our results show that *AtBBX29*, a photomorphogenic and JA-responsive-gene, is a positive regulator of genes involved in the biosynthesis of phenylpropanoids and GSs. Deregulation of *AtBBX29* expression levels alters the accumulation of kaempferol, sinapoyl malate and glucosinolates in leaves, affecting *Arabidopsis* defense responses against the necrotrophic fungus *B. cinerea* and the insect herbivore *S. frugiperda*.

Notable progress has been made in characterizing the roles of plant BBX proteins in many biological processes, such as shade avoidance responses, thermo- and photomorphogenesis, and photoperiodic regulation of flowering [27, 56–60], but the role of BBXs in the photoregulation of specialized metabolites with potential effects on plant defense is poorly understood. Here we show that AtBBX29 is a light-regulated TF with a nuclear localization (Fig. 1e). Its photomorphogenic regulation occurs at the transcriptional level (Fig. 1a), downstream of phytochrome, cryptochrome and UVR8 photoreceptors (Fig. 1b, c). Our data indicate that AtBBX29 positively regulates the expression of flavonoid biosynthetic genes (Fig. 3a); however, the observation of normal transcript levels of some flavonoid biosynthetic genes in *bbx29-1* mutant plants suggests that other BBX proteins could complement the lack of AtBBX29 for the regulation of these genes. Additionally, AtBBX29 induces *MYB12* transcription in *Arabidopsis* adult plants (Fig. 3b) and the transient expression of *AtBBX29* activates the promoter of *MYB12* in *Nicotiana benthamiana* (Fig. 3c). Taken together, these results place AtBBX29 function up-stream of MYB12, with AtBBX29 acting as a positive regulator of flavonoid biosynthetic genes in *Arabidopsis* leaves. This role of AtBBX29 in adult plants contrasts with the one described for the seedling stage, where it has been proposed that AtBBX29 acts as a negative regulator of photomorphogenesis [44, 61]. Variations in the apparent functionality of BBX proteins have been reported before; for example, AtBBX31 negatively regulates seedling photomorphogenesis under white light, but it promotes the accumulation of specialized metabolites in response to UV-B radiation [47].

*Arabidopsis* mutants with defects in the biosynthesis or metabolism of soluble phenolic or GS compounds exhibit an enhanced susceptibility to the attack of pathogens and/or herbivores [29, 30]. Here we show that *bbx29* mutant plants have reduced accumulation of soluble phenolic compounds in their leaves (Fig. 2a), including reduced levels of kaempferol glucosides and sinapoyl malate (Fig. 2b) compared to Col plants. In addition, the concentration of indolic GSs, represented by I3M, was also reduced in *bbx29-1 and bbx29-2* mutant plants in comparison to the wild-type, while 4MSOB or 3MSP accumulation was only significantly reduced in the *bbx29-1* null but not in the *bbx29-2* knockdown mutant (Fig. 5c; SI Fig.S4). In agreement with the reduced GS levels, the *bbx29* mutation negatively affected the expression of *MYB34 and MYB51* genes, the main TFs known to regulate indolic GS biosynthesis in *Arabidopsis* (Fig. 4b). Finally, variations in the accumulation of phenolic compounds and indolic GSs resulting from manipulation of the leaves of *AtBBX29* expression were reflected in the bioassays with the *B. cinerea* and *S. frugiperda*. *bbx29* mutant plants exhibited higher susceptibility to *B. cinerea* (Fig. 5a) and sustained higher caterpillar growth than wild-type plants (Fig. 5c), whereas the opposite was true for *BBX29ox* plants (Fig. 5b, c).

There is increasing evidence that besides their photomorphogenic functions, BBX proteins also play integral roles in several hormone signaling pathways in plants [57, 61, 62], Vaishak et al. [63] showed that AtBBX29 integrate photomorphogenesis and brassinosteroid signaling in *Arabidopsis* seedling development and previous studies suggested that other BBX proteins could play a role in the JA-mediated responses [25, 64]. Here we found that At*BBX29* transcript levels were transiently up-regulated by MeJA (Fig. 6a), but *bbx29* mutant plants had wild-type levels of JA metabolites and JA-marker transcript, both under basal conditions or after MeJA treatment (Fig. 6b, c). Taken together, our results suggest that the main function of AtBBX29 in *Arabidopsis* resistance to attackers is related to its positive effect on the accumulation of defense-related specialized metabolites (kaempferol, sinapates and GSs) in leaves, without interfering with JA metabolism. However, more efforts are needed to characterize the possible roles of AtBBX29 in JA signaling. We also looked for possible homologous of *AtBBX29* in other plant species, but AtBBX29 sequence comparisons against green plant genomes did not reveal any orthologous, although paralogs are present in *Arabidopsis* [43]. It will be a challenge to assess whether (and which) BBX proteins fulfill the role of AtBBX29 in the photomodulation of defenses in other plant species.

UV-B radiation plays an important role in the accumulation of phenolic compounds, which can act as effective sunscreens and also increase plant resistance to a variety of consumer organisms [30, 36, 65–68]. Considering that UV-B radiation strongly induced *AtBBX29* gene expression in a UVR8-dependent manner (Fig. 1a, c), we hypothesized that the increase in resistance against pathogens triggered by low doses of UV-B radiation could be mediated by the action of AtBBX29. The *B. cinerea* bioassay data presented in Fig. 7 are consistent with this hypothesis and suggest that AtBBX29 plays an important role in integrating UV-B signaling pathways with defense responses triggered by UV-B exposure.

## Materials and methods

### Plant material

The following *Arabidopsis thaliana* mutants were used: *bbx29-1* (N638034/SALK_138034) and *bbx29-2* (N582429/SALK_082429) obtained from the Nottingham *Arabidopsis* Stock Centre (NASC). Both lines are in Col-0 background and they were genotyped to obtain homozygous lines and finally checked by RT-qPCR (SI Fig. S1A). *phyA phyB cry1 cry2* quadruple mutant and *uvr8-6* single mutant have been described previously by Favory et al. [69] and Mazzella et al. [70]. To generate transgenic lines over-expressing *Arabidopsis BBX29*, the coding sequence of *BBX29* was amplified by PCR from cDNA using specific primers (Supplemental Table S1). The cDNA was cloned into the pDONR221 plasmid and inserted into the pEarleyGate 201 vector (HA-BBX29 */ BBX29ox*) or pEarleyGate 104 (YFP-BBX29) under the control of the CaMV 35S promoter using Gateway technology (Invitrogen, http://www.invitrogen.com).

### Growth conditions

Plants were grown under short days (10 h light/14 h dark, 18–20 °C, humidity 50–60%) and 110 μmol m^−2^ s^−1^ of photosynthetically active radiation (PAR) provided by LED bulbs (Phillips LED Ecofit T8). For UV-B treatments, white light LED tubes were supplemented for 5 h with UV-B narrowband lamps (Phillips PL-S 9W/01/2P) which delivered photomorphogenic UV-B fluence rates (1 μmol m^−2^ s^−1^) during 5 days. Except indicated otherwise, rosette-stage plants of similar age (3–4 weeks old) and size were selected for the experiments and randomly assigned to treatments. For seedlings experiments, sterilized seeds were sown in clear plastic boxes on 0.8% agar with half-strength Murashige and Skoog medium and incubated in darkness at 4 °C to reduce dormancy and homogenize germination. After 3 days, imbibed seeds were exposed to a red light pulse and incubated in darkness for 24 h at 25 °C to induce germination. Then, the boxes were transferred to their corresponding light treatment in the growth chamber (Percival-Scientific) equipped with red, blue and white light LEDs. PAR and UV-B were measured using an SKP215 PAR sensor and SKU430 UV-B sensor, respectively (Skye Instruments Ltd., Powys, UK).

### Gene expression analyses

Total RNA was extracted using a Spectrum Plant Total RNA kit (Sigma Aldrich) and crude RNA samples were treated with Rnase-free Dnase I according to the protocol (Promega, http://www.promega.com). For RT-qPCR analysis, complementary DNAs were obtained with Superscript III Reverse Transcriptase (Invitrogen) following the manufacturer’s instructions. RT-qPCR analysis was performed on an optical 96-well plate using SYBR Green PCR master mix (ROCHE) and an ABI PRISM 7500 real-time PCR system (http://www.appliedbiosystems.com). *IPP2* (At3g02780) and *UBC* (At5g25760) were used to normalize the expression levels for different concentrations of cDNA. The relative expression levels were calculated using the 2–ΔΔCt method (three pools of three individual plants). Specific primer pairs for each gene are listed in Supplemental Table S1.

### Reporter constructs and transcriptional assays

A *pMYB12::LUC* reporter construct was made by cloning 1387 bp upstream of the start codon of *MYB12* with specific primers (Supplemental Table S1) into the pDONR207 plasmid, and finally recombined into the pGreenII 0800-LUC vector [42, 71] using Gateway technology (Invitrogen). We used *35S::HA-BBX29* as an effector (described above). Transient expression in leaves of 3-week-old *N. benthamiana* was carried out by the infiltration mixtures indicated in Fig. 3c. To prevent silencing, *A. tumefaciens* C58 carrying a construct that expresses the silencing suppressor P19 was included in all the mixtures. Firefly and the control Renilla–LUC activities were assayed from leaf extracts collected 2 days after infiltration with the Dual-Glo Luciferase Assay System (Promega) and quantified with a GloMax 96 Microplate Luminometer (Promega).

### Confocal

*35S::YFP-BBX29* 6-d-old seedlings were transferred to glass slides and analyzed with an LSM 780 confocal laser-scanning microscope (Zeiss). Excitation of the Yellow Fluorescent Protein (YFP) was performed at 514 nm and the emission was detected between 525 and 561 nm.

### Protein isolation and Western blot

Total protein was extracted from 6 d-old seedlings with buffer containing 50 mM Tris pH 7.6, 150 mM NaCl, 10% glycerol, 5 mM MgCl_2_, 0.1% NP-40, 1% DTT, 1% protease inhibitor cocktail (Sigma). The samples were boiled for 10 min in SDS-PAGE buffer, separated by electrophoresis in 10% SDS–polyacrylamide gels and electrophoretically transferred to PVDF membrane, according to the manufacturer’s instructions (Bio-Rad). We used anti-HA (MMS-101R, Covance) and anti-ACT2 (A0480, Sigma) as primary antibodies, and horseradish-peroxidase-conjugated anti-mouse (P0447, Dako) as the secondary antibody. Signal detection was performed using the Amersham ECL Select Western Blotting Detection Reagent (RPN 2235, GE Healthcare) and the Image Quant LAS 4000 mini CCD camera system (GE Healthcare).

### Metabolite determination

For determination of total soluble leaf phenolics, leaf samples (two discs per plant, youngest fully expanded leaves) from 4-week-old plants cultivated under controlled conditions were placed in 1.4 mL of a methanol:HCl solution (99:1, v/v) and allowed to extract for 48 h at − 20 °C. Absorbance of extracts was read in a spectrophotometer at 305 nm (UV-1700 series; Shimadzu). The remaining leaf tissue was freeze-dried and stored in a container with silica gel until HPLC analysis. Individual leaf phenolics were determined by HPLC following the protocol described previously by Demkura et al. [36]. According to Yin et al. [72] we name Kaempferol 1: kaempferol-3-O-[rhamnosyl (1- > 2 glucoside)]—7-O-rhamnoside; Kaempferol 2: kaempferol 3-O-glucoside-7-O-rhamnoside; Kaempferol 3: kaempferol 3-O-rhamnoside-7-O-rhamnoside. For GS determinations we used the youngest fully expanded from 4-week-old *Arabidopsis* leaves following the protocol described in Cargnel et al. [42].

### MeJA treatments and determination of jasmonate pools

For MeJA treatment we sprayed 4-week-old plants with 50 µmol of MeJA (Sigma) or mock solutions (0.1% ethanol). Plants were harvested at 0 (mock), 1, 3 or 5 h after MeJA treatment for gene expression analysis or at 6 h after treatment for jasmonate determinations. We used four biological replicates (each consisting of three individual rosettes) for each genotype and treatment combination. Jasmonate analysis was performed by LC–MS/MS as described previously in Fernandez-Milmanda et al. [53].

### *B. cinerea* and *S. frugiperda* bioassays

*Botrytis cinerea* (strain B05) was grown, maintained and collected as described by Demkura et al. [30]. Four leaves of 4-week-old rosettes were inoculated on the adaxial surface with a 5 µl droplet of a spore suspension. Plants were kept in cylindrical chambers made of clear polyester to prevent desiccation. After 48 h, infected leaves were collected and scanned with an HP Scanjett 4500c (Hewlett-Packard). Lesion areas were measured using ImageJ software. For *Spodoptera frugiperda* bioassay, 6-day-old larvae were placed on 4-week-old *Arabidopsis* under growth chamber conditions as described above. The bioassay was performed with four larvae per plant (10–20 plants per genotype). After 5 days, surviving larvae were collected and weighed. Bioassays were repeated at least three times with similar results. For the bioassays testing the effects of UV-B radiation, the UV-B bulbs were maintained off during the day of inoculation and for the duration of the bioassay.

## Supplementary Information

Below is the link to the electronic supplementary material.

**Additional file 1: Figure S1.** A) Transcript levels of *AtBBX29* in Col, *bbx29-1* and *bbx29-2* mutant plants. B) Transcript levels of *AtBBX29* in Col and two independent overexpression lines (*BBX29ox#4* and *BBX29ox#8*). Values are normalized to *IPP2* transcript levels and standardized to Col expression levels. Each bar represents the mean ± SEM (n ≥ 3 biological replicates). Data were analyzed by Student’s t tests, and asterisks indicate significant differences between Col and mutants or transgenic lines (**P < 0.01, ***P < 0.001). **Figure S****2****.** Transcript levels of *MYB11* and *MYB111* in rosette leaves of Col, *bbx29-1* and *BBX29ox* overexpression lines. Values are normalized to *IPP2* transcript levels and standardized to Col expression levels. Each bar represents the mean ± SEM (n ≥ 3 biological replicates). Data were analyzed by Student’s t tests, and asterisks indicate significant differences between Col and *bbx29-1* or *BBX29ox* transgenic lines (*P < 0.05, **P < 0.01, NS, not significant). **Figure S3.** Transcript levels of genes involved in the GS biosynthetic pathway (*MYB14* and *MYB51*) in Col and *BBX29ox* overexpression lines. Values are normalized to *IPP2* transcript levels and standardized to Col expression levels. Each bar represents the mean ± SEM (n ≥ 4 biological replicates). Data were analyzed by Student’s t tests, and asterisks indicate significant differences between Col and transgenic lines (**P < 0.01). **Figure S****4****.** Glucosinolate (I3M, 3MSP and 4MSOB) accumulation in rosette leaves of Col and *bbx29-2* knockdown mutant plants. Each bar represents the mean ± SEM (n ≥ 4 biological replicates). Data were analyzed by Student’s t tests, and asterisks indicate significant differences between Col and *bbx29-2* mutant plants (*P < 0.05, NS, not significant). **Figure S****5****.** *B. cinerea* bioassay in Col, single mutant *hy5-215* (Col background), Ws and double mutant *hy5-ks50/hyh* (Ws background) plants. Values are means ± SEM (n ≥ 25 individual plants). Data were analyzed by Student’s t tests, and asterisks indicate significant differences between Wild-types (Col or Ws) and mutant lines (**P < 0.01); NS, not significant.

**Additional file 2: Table S1.** List of primers used in this study.

## Data Availability

All data generated or analysed during this study are included in this published article (and its supplementary information files).
