# Supplementary material for: AtBBX29 integrates photomorphogenesis and defense responses in Arabidopsis
Source: Photochem Photobiol Sci. 2023 Feb 18;22(6):1475–89. doi: 10.1007/s43630-023-00391-8 (PMC10310583; doi:10.1007/s43630-023-00391-8)
Supplement: Supplementary file 1 — Additional file 1: Figure S1. A) Transcript levels of AtBBX29 in Col, bbx29-1 and bbx29-2 mutant plants. B) Transcript levels of AtBBX29 in Col and two independent overexpression lines (BBX29ox#4 and BBX29ox#8). Values are normalized to IPP2 transcript levels and standardized to Col expression levels. Each bar represents the mean ± SEM (n ≥ 3 biological replicates). Data were analyzed by Student’s t tests, and asterisks indicate significant differences between Col and mutants or transgenic lines (**P < 0.01, ***P < 0.001). Figure S2. Transcript levels of MYB11 and MYB111 in rosette leaves of Col, bbx29-1 and BBX29ox overexpression lines. Values are normalized to IPP2 transcript levels and standardized to Col expression levels. Each bar represents the mean ± SEM (n ≥ 3 biological replicates). Data were analyzed by Student’s t tests, and asterisks indicate significant differences between Col and bbx29-1 or BBX29ox transgenic lines (*P < 0.05, **P < 0.01, NS, not significant). Figure S3. Transcript levels of genes involved in the GS biosynthetic pathway (MYB14 and MYB51) in Col and BBX29ox overexpression lines. Values are normalized to IPP2 transcript levels and standardized to Col expression levels. Each bar represents the mean ± SEM (n ≥ 4 biological replicates). Data were analyzed by Student’s t tests, and asterisks indicate significant differences between Col and transgenic lines (**P < 0.01). Figure S4. Glucosinolate (I3M, 3MSP and 4MSOB) accumulation in rosette leaves of Col and bbx29-2 knockdown mutant plants. Each bar represents the mean ± SEM (n ≥ 4 biological replicates). Data were analyzed by Student’s t tests, and asterisks indicate significant differences between Col and bbx29-2 mutant plants (*P < 0.05, NS, not significant). Figure S5. B. cinerea bioassay in Col, single mutant hy5-215 (Col background), Ws and double mutant hy5-ks50/hyh (Ws background) plants. Values are means ± SEM (n ≥ 25 individual plants). Data were analyzed by Student’s t tests, and a [file 43630_2023_391_MOESM1_ESM.pptx]

## Slide 1
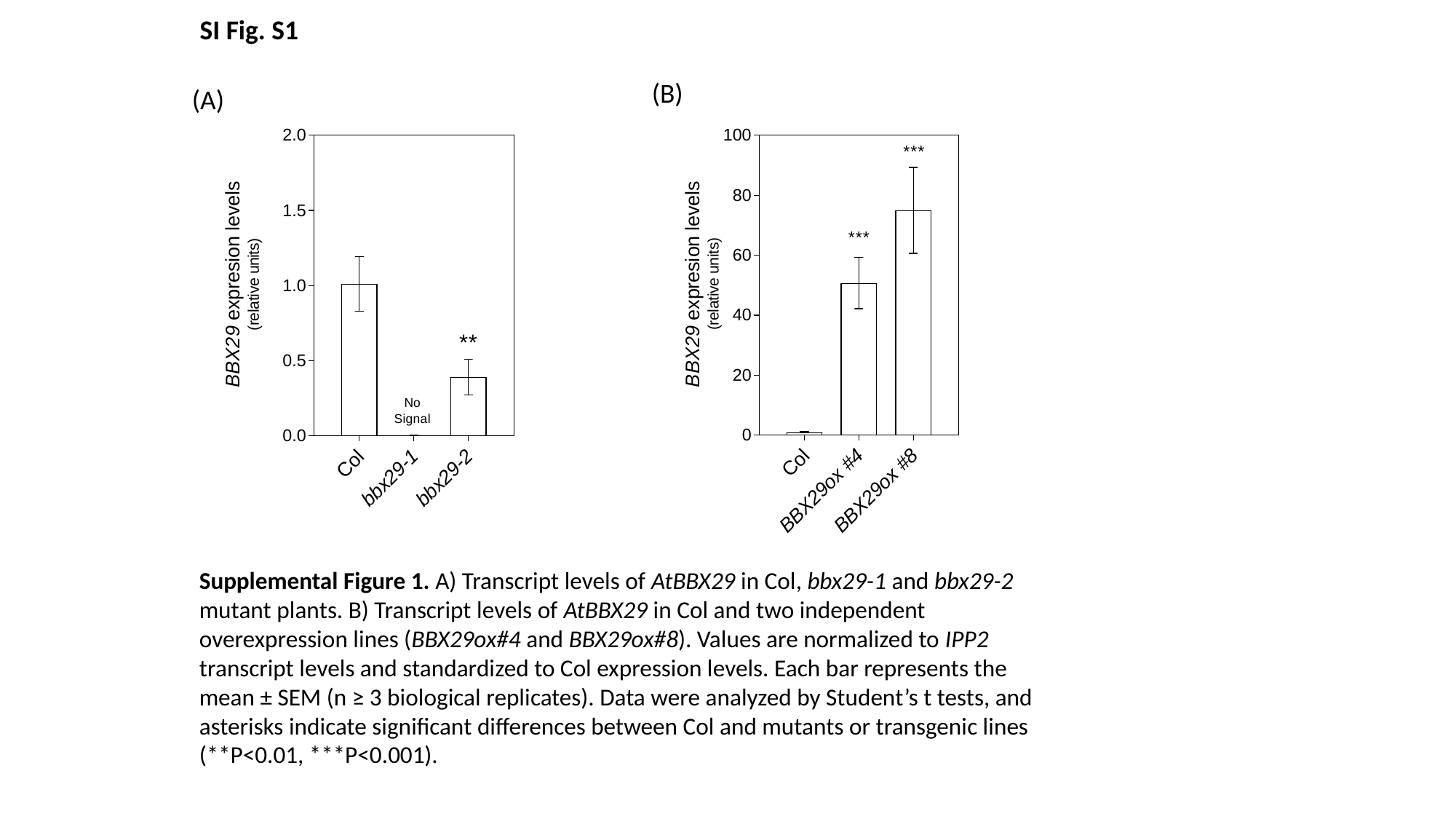

SI Fig. S1
(B)
(A)
Supplemental Figure 1. A) Transcript levels of AtBBX29 in Col, bbx29-1 and bbx29-2 mutant plants. B) Transcript levels of AtBBX29 in Col and two independent overexpression lines (BBX29ox#4 and BBX29ox#8). Values are normalized to IPP2 transcript levels and standardized to Col expression levels. Each bar represents the mean ± SEM (n ≥ 3 biological replicates). Data were analyzed by Student’s t tests, and asterisks indicate significant differences between Col and mutants or transgenic lines (**P<0.01, ***P<0.001).

## Slide 2
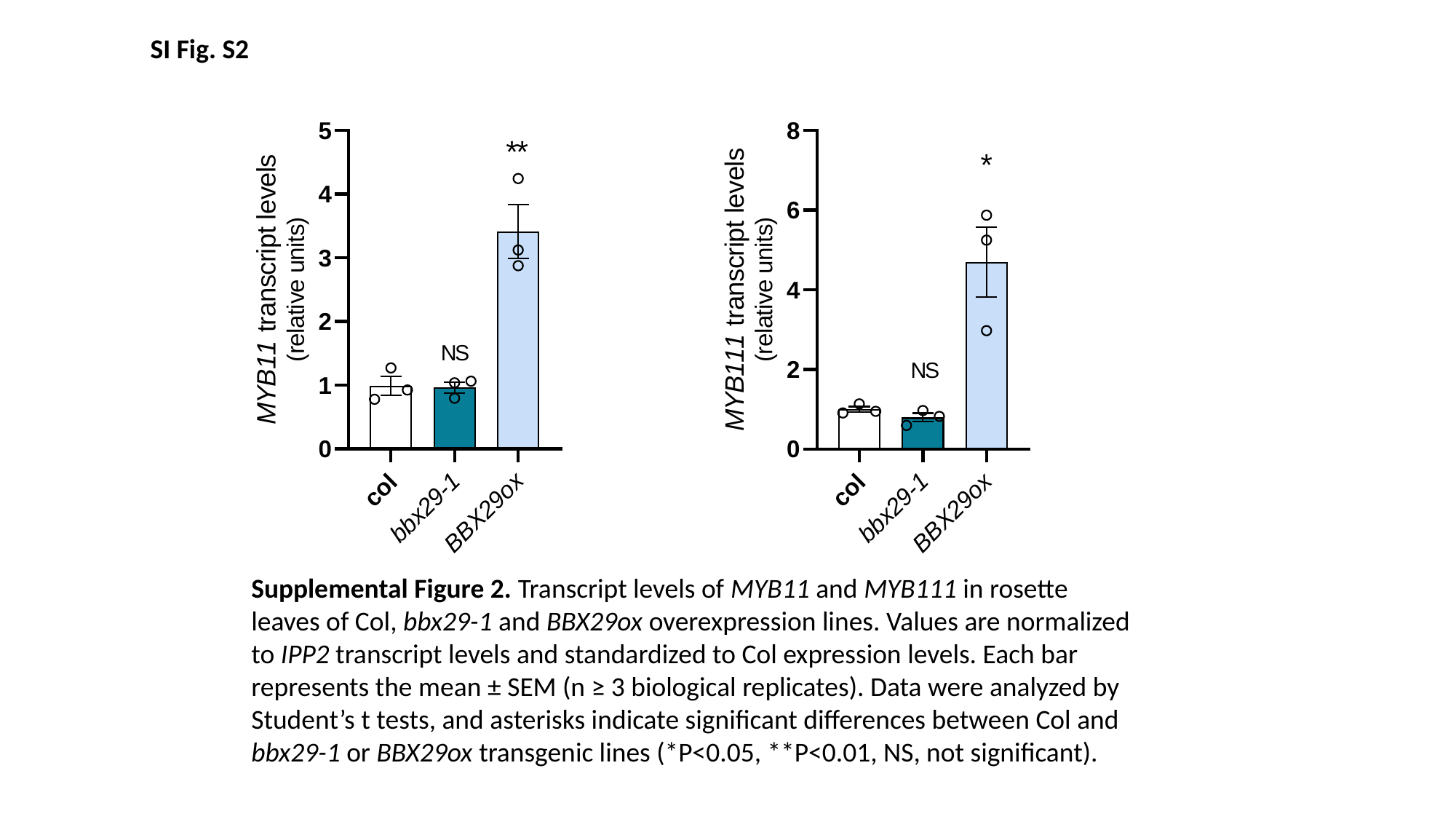

SI Fig. S2
Supplemental Figure 2. Transcript levels of MYB11 and MYB111 in rosette leaves of Col, bbx29-1 and BBX29ox overexpression lines. Values are normalized to IPP2 transcript levels and standardized to Col expression levels. Each bar represents the mean ± SEM (n ≥ 3 biological replicates). Data were analyzed by Student’s t tests, and asterisks indicate significant differences between Col and bbx29-1 or BBX29ox transgenic lines (*P<0.05, **P<0.01, NS, not significant).

## Slide 3
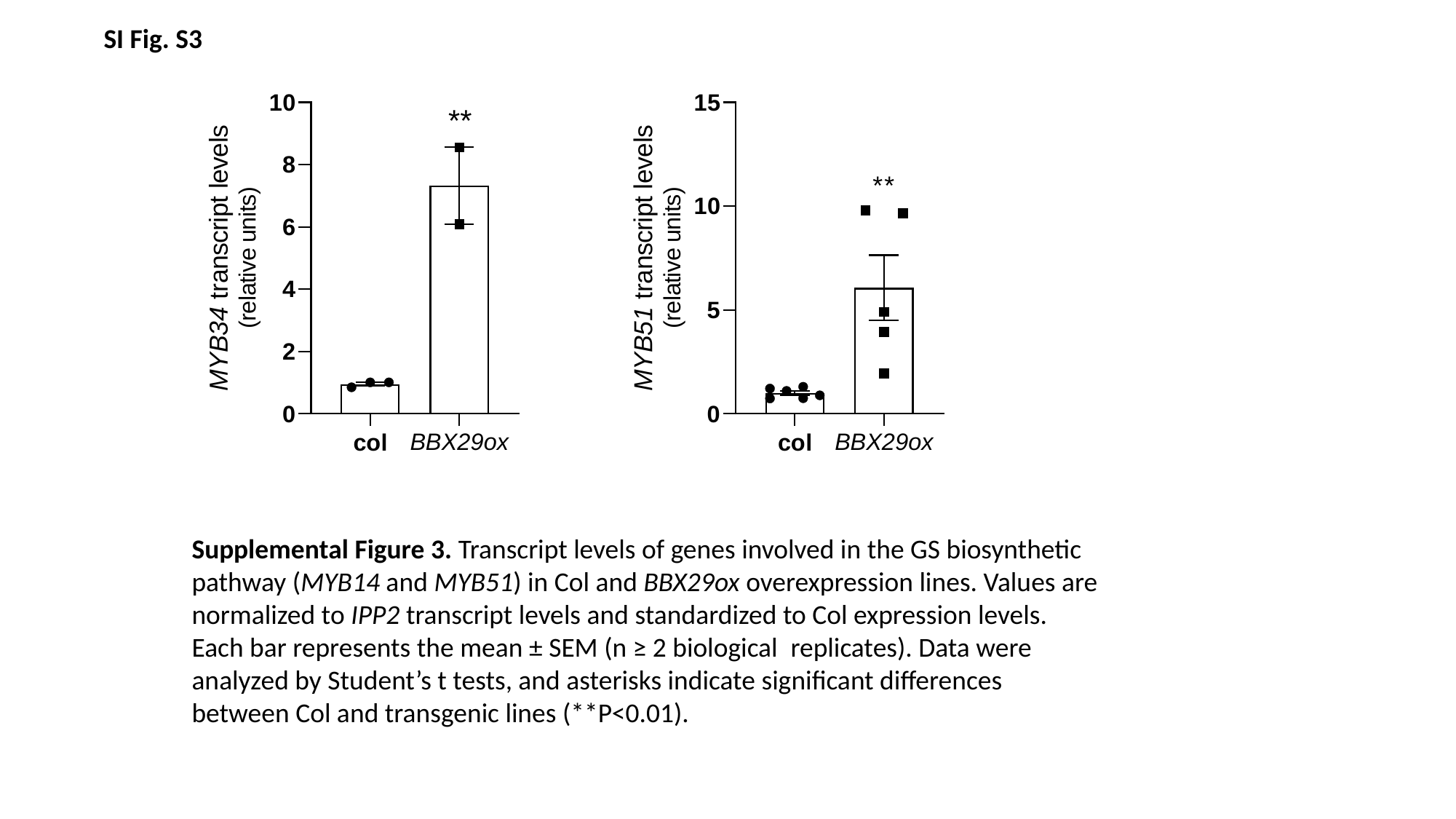

SI Fig. S3
Supplemental Figure 3. Transcript levels of genes involved in the GS biosynthetic pathway (MYB14 and MYB51) in Col and BBX29ox overexpression lines. Values are normalized to IPP2 transcript levels and standardized to Col expression levels. Each bar represents the mean ± SEM (n ≥ 2 biological  replicates). Data were analyzed by Student’s t tests, and asterisks indicate significant differences between Col and transgenic lines (**P<0.01).

## Slide 4
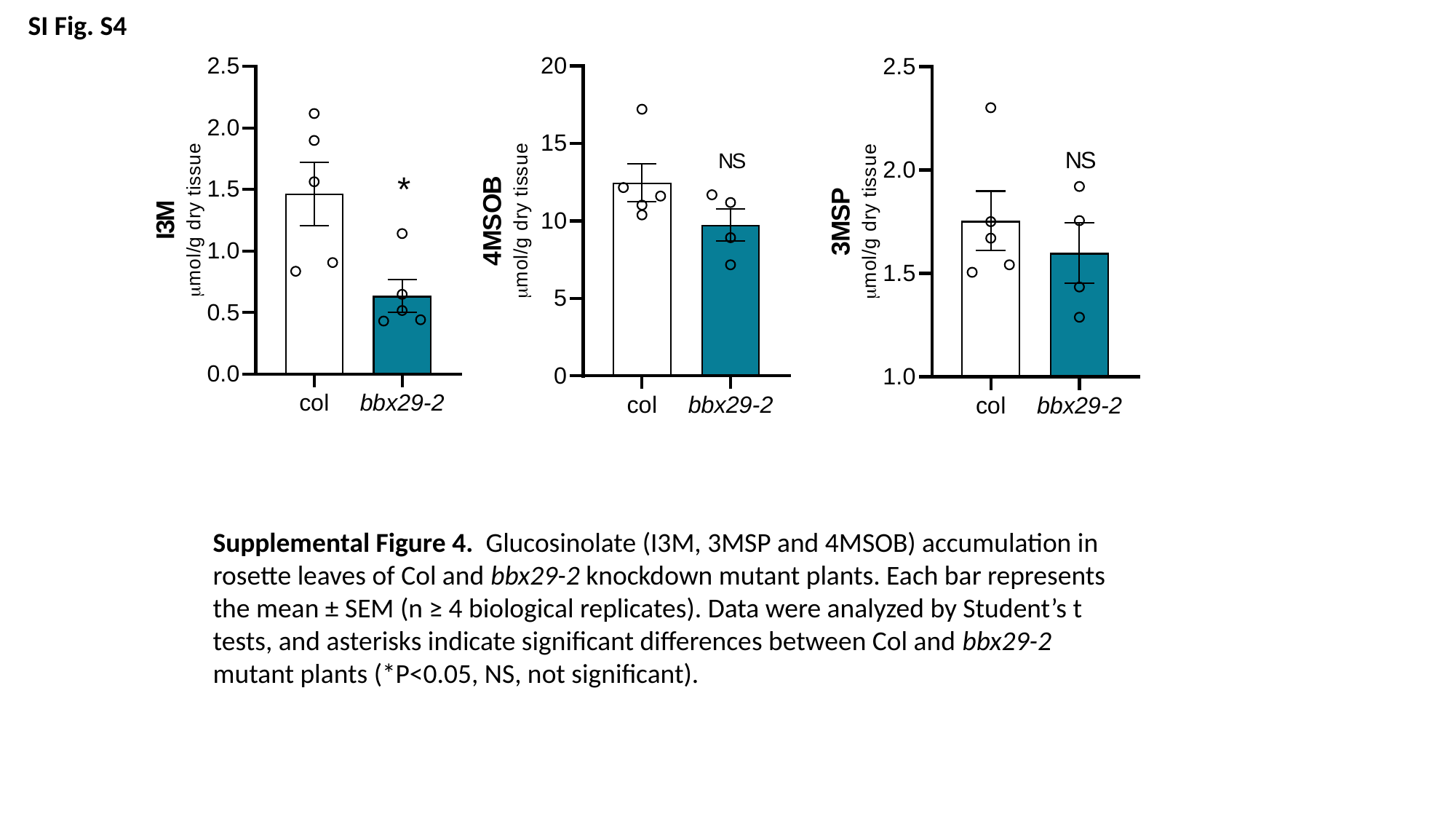

SI Fig. S4
Supplemental Figure 4. Glucosinolate (I3M, 3MSP and 4MSOB) accumulation in rosette leaves of Col and bbx29-2 knockdown mutant plants. Each bar represents the mean ± SEM (n ≥ 4 biological replicates). Data were analyzed by Student’s t tests, and asterisks indicate significant differences between Col and bbx29-2 mutant plants (*P<0.05, NS, not significant).

## Slide 5
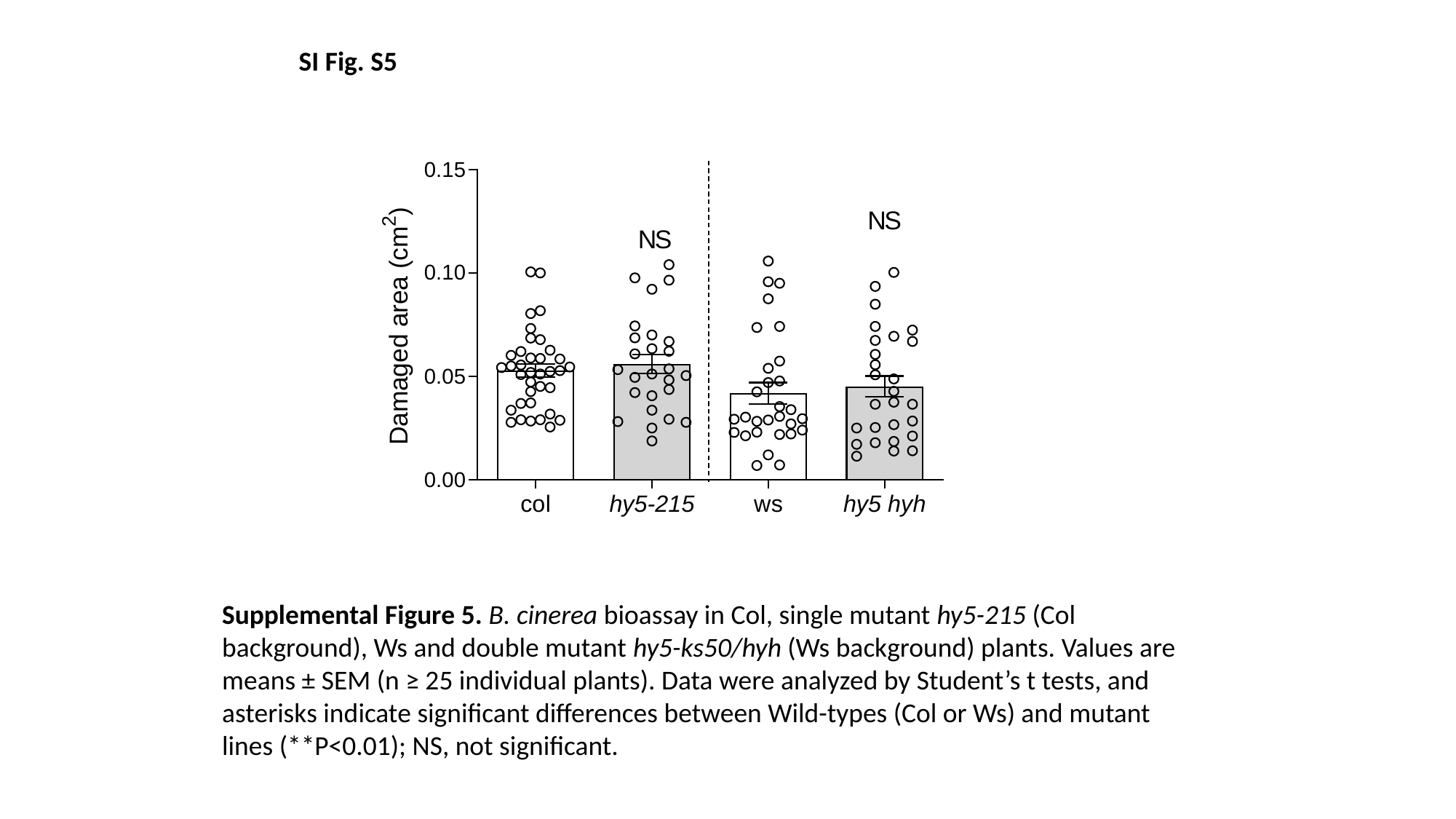

SI Fig. S5
Supplemental Figure 5. B. cinerea bioassay in Col, single mutant hy5-215 (Col background), Ws and double mutant hy5-ks50/hyh (Ws background) plants. Values are means ± SEM (n ≥ 25 individual plants). Data were analyzed by Student’s t tests, and asterisks indicate significant differences between Wild-types (Col or Ws) and mutant lines (**P<0.01); NS, not significant.
